# Zooplankton protect viruses from sunlight disinfection

**DOI:** 10.1128/aem.02540-24

**Published:** 2025-03-31

**Authors:** J. A. Wang, O. Aryal, L. N. Brownstein, H. Shwwa, A. L. Rickard, A. E. Stephens, M. Lanzarini-Lopes, N. S. Ismail

**Affiliations:** 1 Picker Engineering Program, Smith College 6089, Northampton, Massachusetts, USA; 2 Department of Civil and Environmental Engineering, University of Massachusetts Amherst 14707, Amherst, Massachusetts, USA; Colorado School of Mines, Golden, Colorado, USA

**Keywords:** zooplankton, viral inactivation, rotifers, enteric viruses, sunlight disinfection

## Abstract

Sunlight disinfection is an important inactivation process for enteric viruses in water. Understanding how dark biotic processes, such as zooplankton filter feeding, impact sunlight disinfection for viruses has important implications for public health. This research quantifies the uptake of MS2, a model for enteric viruses, by the filter feeder *Branchionus plicatilis* (rotifer) and the effects of such uptake on subsequent sunlight inactivation of MS2. Experiments co-incubating MS2 with rotifers showed 2.6 log viral removal over 120 hours. Viable virus was recovered from rotifer bodies after co-incubation, indicating incomplete viral inactivation via ingestion. When live rotifers were co-incubated with MS2 and the system was treated with sunlight, experimental treatments with rotifers showed that the virus was protected with 2–3 log viral inactivation compared to 4.5 log inactivation for sunlight controls without rotifers. Dead rotifers placed in the system did not show the same magnitude of protection effects, indicating that active filter feeding of rotifers is associated with protection from sunlight. Data from this study show that zooplankton may serve as a vector for viruses and reduce the efficiency of sunlight inactivation.

**IMPORTANCE:**

Enteric viral contamination in water is a leading global cause of waterborne disease outbreaks. Sunlight inactivation is an important disinfection mechanism in natural waters, but accurately modeling inactivation is challenging due to the complex nature of aquatic systems. Zooplankton play a critical role in natural systems and are known to inactivate bacteria, but their interaction with viruses is not well understood. Our research examines the impact of a model zooplankton species on the sunlight disinfection of viruses. The results from this study address knowledge gaps in the importance of dark processes such as zooplankton filter feeding and their impact on viral fate.

## INTRODUCTION

Various abiotic and biotic processes can impact the inactivation of enteric viruses in aquatic systems (1–4). Sunlight-mediated disinfection (light processes) has been shown to play a critical role in the inactivation of enteric viruses in sunlit waters. Sunlight-mediated disinfection, including direct and indirect endogenous and exogenous inactivation, has been studied in depth for enteric viruses, and numerical models have been developed to predict sunlight inactivation rates (3). These processes require the virus or chromophores within or near the virus to absorb sunlight and undergo chemical transformation that damages the ability of the virus to reproduce and infect a host. The rate and extent of light reactions are highly dependent on water quality and solar exposure within a complex water matrix. Therefore, weather, location, water body depth, and physico-chemical water properties significantly affect interaction of light to both virus and chromophores in natural environments (1, 2, 5, 6). Additionally, dark physico-chemical (abiotic) processes can both expedite or impede sunlight inactivation mechanisms. For example, adsorption and sedimentation due to particulate matter can change the rate of light, virus, and chromophore availability in water and have variable effects on sunlight inactivation (1, 6–8). Current sunlight inactivation models that consider each abiotic mechanism in isolation and neglect biotic mechanisms fail to capture the synergistic and antagonistic effects of natural systems.

In contrast to the vast number of studies examining abiotic factors, knowledge gaps exist in understanding the interactions of biotic mechanisms with sunlight disinfection. For example, zooplankton grazing can result in a reduction in viral concentrations in aquatic systems (5, 9–13), but it is unknown if sunlight changes these interactions. In addition to sunlight impacting grazing activity, zooplankton can also potentially impact the efficacy of disinfection by acting as a viral reservoir and protecting viruses from sunlight inactivation. While filter-feeding zooplankton have been shown to uptake a variety of viral types, the fate after uptake is variable (10–16). Some studies have shown that viruses are inactivated by zooplankton upon ingestion, while others have detected viable virus within the organisms or virus excreted by these organisms (10–17). Since zooplankton can potentially uptake viruses without inactivation, they have the potential to hinder sunlight-mediated disinfection and disperse viruses throughout the aquatic system, but further research is needed to explore this hypothesis.

Various studies show that zooplankton protect bacteria from chlorination, ozonation, and UV 254 disinfection (18–22), but there is very limited information on viral protection from disinfection by zooplankton. Only one published study showing that amoeba protect viruses from disinfection exists (23). None of these previously published studies quantify viral uptake rate by rotifers or the effects of their grazing on sunlight disinfection. In order to better predict how dark processes impact disinfection of viruses, our study examines the viral rate of uptake by zooplankton and also quantifies viral protection by rotifers against sunlight disinfection. Our work provides data to help accurately predict overall inactivation in natural systems and to improve existing sunlight inactivation models.

We utilize a model zooplankton species, *Branchionus plicatilis*, which is a brackish water rotifer. This globally distributed rotifer can be found in high abundance in tidal wetlands, temporary saline lakes, and coastal lagoons (24). *B. plicatilis* can dominate zooplankton assemblages and tolerate extreme abiotic conditions (25–27). Due to its ubiquity, high reproductivity rates, and hardiness, *B. plicatilis* has been suggested for use as a bio-tool in various applications related to water quality improvement (24). Freshwater species of rotifers within the *Branchionus* genus can also be found in many aquatic systems and have similar anatomy and feeding approaches. Hence, findings from this one model species could extend to other zooplankton. We utilize the bacteriophage MS2 as a model virus. MS2 is often used as a conservative model virus due to its resistance to sunlight-mediated inactivation in comparison to many human pathogenic viruses and other phages (28–31). Hence, our results will not only address knowledge gaps related to MS2 inactivation but will also be applicable to enteric virus inactivation.

## MATERIALS AND METHODS

### Rotifer culture and preparation for laboratory experiments

Bdelloid rotifers *Brachionus plicatilis* were maintained in 15 ppt brackish water (MilliQ water with Instant Ocean Salt) at 20°C and fed *Nannochloropsis sp*. algae (Reed Mariculture) *ad libitum*. Rotifers were acclimated from 20°C to 15°C within a temperature-controlled environmental chamber (Thermo Scientific Forma) by decreasing the temperature by 1°C every 24 hours in preparation for experimental use. Immediately before the experiment started, rotifers were enumerated and appropriately diluted in 15 ppt saltwater to obtain a final concentration of approximately 200 rotifers/mL, with each beaker containing 300 mL of brackish water. Although rotifer densities vary based on different system conditions (32–35), they can reach very high densities. For example, in waste stabilization ponds, rotifer densities reached 150,000 individuals/liter (36). Hence, the rotifer density chosen for these experiments may be representative of seasonal peaks in rotifer density as well as aquatic environments that typically contain very high densities, such as natural systems used for wastewater treatment. For rotifer enumeration, a sample between 3 and 10 mL was taken from the stock culture, and 25 µL/ml of formaldehyde (Fisher Scientific, 37%) was utilized to euthanize the rotifers. A 1 mL sample was then loaded on a Sedgewick rafter (Wildco) and rotifers were counted using a compound light microscope (Olympus) at 4× magnification. Triplicate samples were counted to obtain an average rotifer count. Algal concentration was counted using a hemocytometer as well as a Z2 Beckman Coulter Counter. The algal spike amount for experimentation was determined based on feeding studies in which the algal clearance rate was calculated and rotifer mortality and swimming behavior were observed over 7 days.

### MS2 propagation and enumeration

MS2 (ATCC 15597-B1) was replicated in *Escherichia coli* F_amp_ host (ATCC 700891). Phage propagation and purification were completed using an adapted previously published protocol (37). Briefly, bacterial cells were lysed using chloroform, and debris was removed by centrifugation at 4,000 × g for 15 minutes, followed by vacuum filtration through a 0.22 µm nitrocellulose filter. The filtrate containing MS2 was purified further using 100 kDa filters (MilliporeSigma) by transferring 50 mL of the solutions to the filters and centrifuging at 3,000 × g for 30 min. Subsequently, 50 mL of phosphate buffer solution (PBS, Fisher Chemicals) was added, followed by centrifugation. Finally, the purified viral stock was then eluted at 1,000 × g for 2 minutes. The final virus stock solutions were sterile-filtered through 0.2 µm syringe filters. Purified stocks were stored at −80°C until use. Bacteriophages were assayed using the double agar overlay (DAL) method with 100 µL of sample (38, 39). Plates were incubated at 37°C between 18 and 24 hours, and plaques were counted. Results were recorded as plaque-forming units per milliliter (PFU/mL). Samples were serially diluted using PBS to achieve plate counts between 30 and 300 PFUs. The LOQ for MS2 was 300 PFU/mL, which is calculated based on a detection limit of 30 PFU per 100 µL sample. Controls of bottom agar, top agar, and top agar containing *Escherichia coli* F_amp_ host were plated and incubated.

### Rotifer and MS2 co-incubation experiments

Rotifers were co-incubated with MS2 to quantify uptake rates of the virus. Batch microcosms consisted of aerated beakers containing 300 mL of 15 ppt brackish water spiked with 100–200 µL of MS2 to reach an initial titer in solution of ~10^6^ PFU/mL. The three experimental beakers contained 180 ± 30 rotifers/mL and fed 200 µL of *Nannochloropsis sp*. algae each day of the experiment to provide an adequate food source to maintain the rotifer culture. A control beaker, without rotifers, contained an equivalent MS2 spike as experimental beakers (~10^6^ PFU/mL) and an initial algae spike of 60 µL in brackish water. The algal spike amounts represent the concentration of algae needed to sustain rotifers as well as the calculated residual algal concentration observed at the end of 24 hours of rotifer feeding. Algae were added to the control beakers to have a comparable matrix to the experimental beakers with the primary difference being the absence of rotifers. Every 24 hours, water was sampled, filtered through a 0.22 µm filter to remove rotifers and algae, and enumerated for MS2. In addition, a 3 mL rotifer sample was collected from each beaker for rotifer enumeration. Co-incubation experiments were conducted for up to 120 hours. In all subsequent portions of the paper, rotifer beakers, which were co-cultured with MS2 prior to sunlight inactivation experiments and acclimated to MS2, are referred to as primed rotifers.

### Sunlight exposure experiment

#### Experimental outdoor setup

The experiment setup was deployed outdoors on the top floor of a three-story building for 9.5 hours in Massachusetts. The two experiments presented in this paper started at 8:45 a.m. and 8:48 a.m., respectively. Images of the setup are available in the supplemental material (Fig. S1). Beaker microcosms were fitted into a water bath (Sterilite 33” × 18” × 7”) maintained at 15°C by running chilled water using a chiller (Isotemp) through ¾” copper tubing (Fig. S2). Submersible pumps were set up in the water bath to further circulate the water and ensure a uniform temperature. Water temperature was recorded continuously throughout the duration of the experiment using a thermocouple (Omega 4-Channel Datalogger Thermometer). In addition, the temperature was manually noted at each sampling time point to ensure that it did not deviate more than 5^o^C due to solar heating. Each microcosm consisted of an 800 mL beaker wrapped in black electrical tape to ensure that sunlight could not enter through the sides of the beakers. Fifteen beakers were then placed in the water bath that was fitted with a transparent acrylic sheet containing circular openings to keep each beaker in place. The openings of the dark control beakers were covered with foil to exclude light, and all other beakers were covered with clear plastic film. The transparent plastic film covering each beaker was tested using a spectrophotometer to confirm that it did not impact the measured sunlight spectra. Chemical actinometry was used to measure the light absorbed by the solution during each of the experimental configurations. The reactor setup was replicated in the chemical actinometry runs; therefore, any optical changes due to configuration are accounted for.

Since the experiment was conducted outdoors, there was variability in the irradiance throughout the day as well as between the two experimental days (Fig. S3 and S4). The middle of the day had a higher overall irradiance compared to the beginning and end of the day. During the second experimental day, more clouds were visually observed, which corresponds to the lower irradiance at different experimental time points (Fig. S3 and S4).

Water from each beaker was sampled every 90 minutes for use in MS2 enumeration. Samples for MS2 enumeration were immediately filtered through a 0.22 µm syringe filter (Millipore Sigma Aldrich). Unfiltered samples were also taken at the same time intervals to determine absorbance values of the different solutions. To account for light scattering associated with particles in the solution, we took absorbance measurements with a UV-vis spectrophotometer with an integrating sphere (Thermo Fisher). Additional unfiltered samples were taken at the start and end of the experiment to obtain rotifer counts and algal cell counts. All beakers were aerated, resulting in uniform mixing of the solutions.

#### Live rotifer experiment setup

The initial microcosm algal and MS2 concentrations were equivalent to those detailed in the co-incubation experiments. Experimental microcosms (in triplicate) containing rotifers were exposed to sunlight using the setup detailed above (Fig. S1 and S2). Two different rotifer treatments were tested. Primed rotifers were pre-exposed to MS2 using the setup detailed in the co-incubation experiments with 72 hours of co-incubation with MS2. Naïve rotifers were acclimated to 15°C but were not pre-exposed to MS2 before the sunlight exposure experiment. Sunlight exposed light control beakers (triplicate) were included. In addition, individual dark controls consisted of naïve rotifers, primed rotifers, and no rotifers. Fig. S2 shows an example schematic of the setup.

#### Dead rotifer experiment setup

Dead rotifers were prepared by freezing rotifers in centrifuge tubes on dry ice and ethyl alcohol (Pharmco, 200 proof) bath contained within a Styrofoam cooler for 20 minutes. Immediately after freezing, the rotifers were then placed in a 30°C water bath for 25 minutes. This procedure resulted in dead rotifers with intact bodies. The dead rotifer experimental setup was identical to what is detailed above with the following microcosms exposed to sunlight: triplicate beakers of live naïve rotifers, triplicate beakers of dead rotifers, and an individual sunlight control without rotifers. Individual dark controls consisted of live naïve rotifers, no rotifers, and dead rotifers. Rotifers were examined using a compound light microscope at the start and end of the experiment to ensure that bodies remained intact.

### Rotifer tissue analysis

The concentration of MS2 in rotifer tissue was determined by blending the rotifer bodies, filtering, and plating the resulting solution. The contents of each rotifer beaker from the co-incubation as well as sunlight exposure experiments were poured through a 53 µm sieve, and the water containing residual MS2 in solution was discarded. Rotifers were rinsed from the sieve into a sterile 50 mL centrifuge tube using PBS to remove any MS2 attached to the rotifer bodies. For each rinse cycle, the rotifer and PBS solution were vortexed in the 50 mL centrifuge tube for 2 min to dislodge any sorbed MS2 from the body. The sieving, rinsing, and vortexing procedure was repeated three times. The above protocol resulted in intact rotifer bodies that were not ruptured as a result of vortexing.

To remove the remaining PBS from the rinse, we completed membrane filtration using pre-weighed, pre-wet 50 µm sterile nylon filters. After the wet rotifer weight was obtained, the rotifers were dislodged from the filter by placing the filter in a sterile centrifuge tube filled with 25 mL of PBS, using sterile tweezers. The centrifuge tube was mixed with a vortex for 30 seconds, and the contents were allowed to settle for 15 minutes. The filter was removed using a sterile pair of tweezers and checked under a microscope to confirm the complete removal of rotifers from the filter. The centrifuge tubes containing the rotifers and PBS were centrifuged at 3,200 × g for 30 minutes to form a pellet. After centrifugation, the supernatant was removed, and the rotifer pellet was transferred to a new microcentrifuge tube for an additional centrifuge cycle at 21,000 × g for 10 minutes. After centrifuging, the resulting rotifer pellet in each centrifuge tube was blended using a pellet pestle motor (Kimble Kontes) for 2 minutes. The tissue sample was serially diluted in PBS and plated to determine MS2 concentration. The above rinsing and enumeration procedure was tested with dead rotifer bodies to confirm that the rinsing procedure removed any sorbed virus and ensure the values obtained represent the concentration of MS2 within the bodies of live rotifers.

### Chemical actinometry

A p-nitroanisole (PNA) (Acros Organics 4-Nitroanisole 99%+) and pyridine (pyr) (Fisher BioReagents) chemical actinometer was prepared according to previously published methodology (40, 41). A fresh actinometer solution was made at the start of each experiment and run simultaneous with the light exposure experiments. The actinometer setup was positioned next to the light experiments to absorb the same amount of light. Briefly, 0.01 M PNA solution was prepared in HPLC-grade acetonitrile (ACN) and 1 mL of the 0.01 M PNA solution was transferred to 999 mL HPLC-grade water (Fisher Chemical). Pyridine (15.9 mM) was added to the PNA solution, and the solution was thoroughly mixed. Any remaining solution was then immediately covered in foil and stored in the dark at 4°C for use during the same experiment. Actinometer samples were taken every 45 minutes (1 mL) and placed in 2 mL screw thread autosampler amber vials (Fisher Scientific) for analysis and immediately stored in a 4°C fridge to prevent chemical degradation. All actinometer samples were analyzed within 24 hours of collection, and actinometer solutions and samples were stored in the dark. The dark stored actinometer solutions were tested to confirm that the solution had not degraded with time and changes detected with the actinometer could be attributed to sunlight.

A Shimadzu high-performance liquid chromatography system (HPLC) with a Discovery HS C_18_ 5 µm column (Supelco) was used for actinometer sample analysis. A mobile phase containing 1:1 acetonitrile and HPLC-grade water was utilized, and 80 µL of each sample was run in triplicate at a flow rate of 1 mL/min for 9 minutes. Standards ranging in concentration from 0.976 μM to 62.5 µM of p-nitroanisole (PNA) were made using the remaining 0.01 M PNA + ACN stock solution described above to create a calibration curve. Peaks at 300 nm were integrated to calculate concentrations of the experimental actinometer samples. The PNA concentrations from the HPLC were utilized in conjunction with flux values obtained from a spectrophotometer (Avantes). The measurement of flux values from the spectrometer for the wavelength range of 300–370 nm (wavelength range absorbed by PNA) was obtained every 45 minutes to coincide with actinometer sample times. The actinometer solution absorbance was also obtained using a UV-vis spectrophotometer (Thermo Scientific). Before the start of sunlight exposure experiments with organisms, the actinometer was tested in all 15 spots to confirm that the sunlight exposure was uniform.

### Data analysis

All statistical analyses were completed using SPSS (v28, IBM). The uptake rates (*k*, hr^−1^) for the rotifers from co-culture experiments were calculated by fitting the entire experimental time series to a log-linear model with a shoulder (42):


(1) $$C_{t}=C_{o}e^{-kt}\left[\frac{e^{kS}}{1+\left(e^{kS}-1\right)e^{-kt}}\right]$$

where *C_o_* is the MS2 concentration at *t* = 0 hours in PFU mL^−1^, *C_t_* is the MS2 concentration at a given time point in PFU mL^−1^, *t* is time in hours, *k* is the uptake rate in hours^−1^, and *S* is the shoulder length in hours (lag time). Equation 1 was used to calculate *k*-values for experimental microcosms with rotifers and control microcosms without rotifers. The net uptake rate (*k*_net_, hr^−1^) was calculated as follows:


(2) $$k_{net}=k_{experimental}-k_{control}$$

The net log removal was also calculated from co-culture experiments as follows:


(3) $$log{\left(\frac{C}{C_{o}}\right)}_{net}=log{\left(\frac{C}{C_{o}}\right)}_{experimental}-log{\left(\frac{C}{C_{o}}\right)}_{control}$$

For sunlight exposure experiments, inactivation rate constants were calculated with respect to time (*k*, hr^−1^) and fluence (*κ*, m^2^ kJ^−1^) based on a first-order decay model:


(4) $$C_{t}=C_{o}e^{-kt}$$

(5) $$C_{t}=C_{o}e^{-\kappa F}$$

where *C_o_* is the MS2 concentration at *t* = 0 hours in PFU mL^−1^, *C_t_* is the MS2 concentration at a given time point in PFU mL^−1^, *t* is time in hours, and *F* is the fluence in kJ m^2^.

Before calculating the fluence, the irradiance values were corrected for light absorbance by the rotifers and algae using the correction (screening) factor in Equation 6 (3, 29). The absorbance spectrum (α_s_) of each solution was obtained using a UV-vis with an integrating sphere to account for light scattering by the rotifers and algae (Fig. S5).


(6) $$\left\langle I_{avg}\left(z,\lambda \right)\right\rangle =I_{0}\left(0,\lambda \right)\left(\frac{1-10^{-\alpha_{s}\left(\lambda \right)z}}{2.303\alpha_{s}\left(\lambda \right)z}\right)$$

where $\left\langle I_{\text{avg }}(z,\lambda )\right\rangle$ in W cm^−2^ nm^−1^ is the spectral irradiance averaged over the volume of the experimental solution in each microcosm, $I_{0}(0,\lambda )$ is the absolute incident spectral irradiance in W cm^−2^ nm^−1^, α_s_ (λ) is the absorbance spectrum of the experimental solutions in cm^−1^, and *z* is the depth of the experimental solution in each microcosm (path length) in cm.

Analysis of variance (ANOVA) and analysis of covariance (ANCOVA) were performed to test for differences among treatments and control groups. Spearman's rank order correlation was used to test for relationships between variables. Results were considered significant for *P* < 0.05 for all statistical analyses. All MS2 concentration data were log-transformed before statistical analysis. The mean ± standard deviation (SD) or propagated error values using standard deviation (SD) are presented for experimental data. Both standard deviation and propagated error are referred to as SD in the manuscript. For calculated rate constants (*k*-value and *κ*-values), the standard error (SE) is reported.

## RESULTS AND DISCUSSION

### Rotifers remove virus from solution via filter feeding

**Fig 1:**
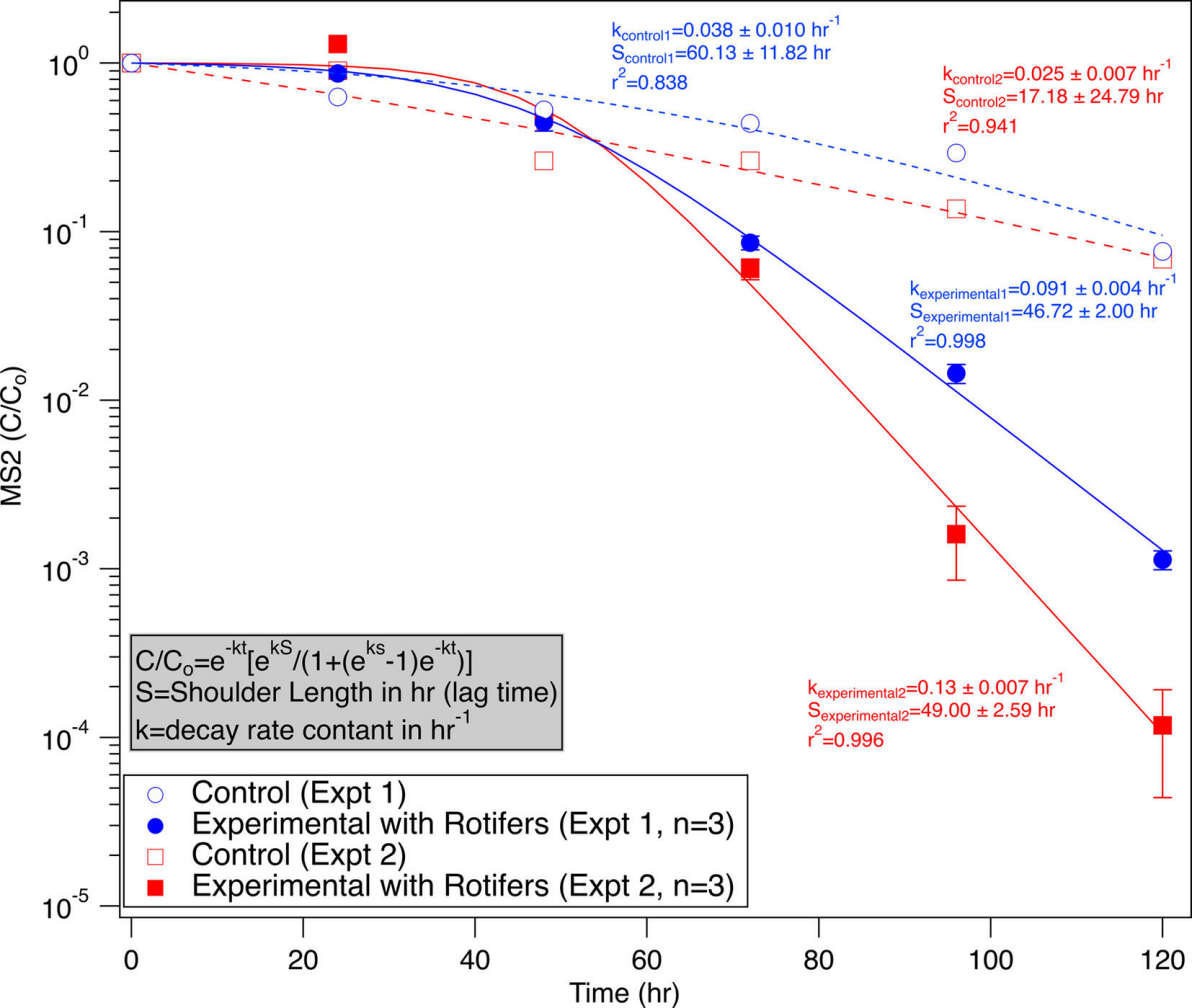
Viral removal by rotifers in two different batch experiments. A log-linear fit with a shoulder was utilized to calculate removal rate constants (*k*-values). Experimental treatments were completed in triplicate, and error bars represent the propagated error for triplicate treatments. Blue circles and red squares correspond to Experiments 1 and 2, respectively.

Rotifers co-incubated with MS2 for 120 hours showed an average net log removal of 2.6 ± 0.08. Fig. 1 shows time series data from two 120-hour co-incubation experiments, each containing experimental rotifer microcosms in triplicate and control microcosms without rotifers. A log-linear model with a shoulder was fit to both the control and experimental data for consistency (Eq. 6). However, a log-linear equation would also fit the control data.

These experiments showed consistent removal of MS2, and there was no significant variability in the net log removal observed in the various experimental microcosms between batches of rotifers (*P* > 0.05). There was no significant relationship between rotifer concentration and log removal values (*P* > 0.05). Additional co-incubation experiments, with shorter exposure duration, were completed for methodological development. For 96-hour co-incubation experiments, a net log removal of 1.6 ± 0.24 was observed (*n* = 11, log removal ranging from 1.58 to 2.7). Despite the variability in net log removal, rotifers consistently removed MS2 from water compared to the control microcosms. When comparing data from multiple experiments with co-incubation times of 96 and 120 hours, no significant relationship was found between the concentration of rotifers at the end of the experiment and the net log removal of MS2 (*P* > 0.05).

As this is the first study to quantify the uptake rates of MS2 by rotifers, our rate constants (*k*_net,expt1_= 0.07 hr^−1^ and *k*_net,expt2_=0.1 hr^−1^) cannot be directly compared to other studies. However, studies examining the interactions between other zooplankton species and viruses do exist, but results on uptake and fate can vary dramatically based on zooplankton and virus type (9–11, 13, 16, 17, 23, 43–45). For example, studies with the ciliates *Tetrahymena pyriformis* show net removal values from less than 1 to nearly 3 log over 48 hours, depending on the virus studied, with 0.7 log net removal specifically for MS2 (10). A study of *Tetrahymena thermophila*, with a similar experimental setup but a different incubation solution, found 1.1 log removal. However, net log removal in this study with *T. thermophila* approached 0.5 when considering the decline observed in the control (12). Other studies have reported ingestion of viruses in amoebae as well (17, 23). For the rotifers, used as a model organism in our study, net log removal of MS2 varied from 1.8 to 2.8 after 120 hours, but no difference between control and experimental treatments was observed at 48 hours. Therefore, log removals are not directly comparable with these different organisms. While it may seem that rotifer uptake of viruses is slower than that of *Tetrahymena sp*., the concentration of *Tetrahymena sp*. used in previously published experiments was two orders of magnitude greater than that of rotifers. The rotifer concentration used in this study is most representative of peak rotifer densities or densities in systems used to treat wastewater, and rotifer density can vary orders of magnitude depending on system conditions (46–48). Despite the potential difference in rotifer densities, the observed uptake of MS2 by rotifers indicates that they can have an impact on water quality and microbial communities.

### Ingestion of virus by rotifers contributes to protection against sunlight inactivation

MS2 inactivation rates in the control treatments without rotifers were first calculated and compared to other published literature values (*k* = 1.3 ± 0.07 hr^−1^ or *κ* = 0.058 ± 0.02 m^2^ kJ^−1^). MS2 was inactivated in brackish water that contained algae, so the rates were higher than those observed in other studies using freshwater, clear seawater, or PBS (28, 29, 49). Increased water salinity has been shown to increase sunlight inactivation of F-RNA phages, and many other environmental factors can also impact phage survival (31, 50). The presence of algae in our controls may have contributed to the exogenous inactivation of MS2 (51), resulting in higher inactivation rate constants. In addition, a direct comparison of rate constants with respect to time is not possible since time-based *k*-values do not take into account differences in irradiance and cumulative fluence. While some studies report fluence-based *κ*-values, the wavelength ranges used when calculating the cumulative fluence vary, which further complicates comparison of rate constants. The UVB range (290–320 nm) is considered to be the primary contributor to inactivation of MS2 for endogenous inactivation, and studies have shown that nucleic acids and proteins of waterborne nonenveloped viruses (like MS2) are not impacted by wavelengths above 320 nm (28, 29, 49). However, the UVA (320–400 nm) range can also contribute to indirect exogenous inactivation, especially when particulate matter such as algae exists in a system (51). In our study, due to the algae present in our system, we used chemical actinometry to obtain cumulative fluence and calculated fluence for the wavelength range covered by PNA-PYR actinometry (300–370 nm) that spans the UVB and UVA ranges.

**Fig 2:**
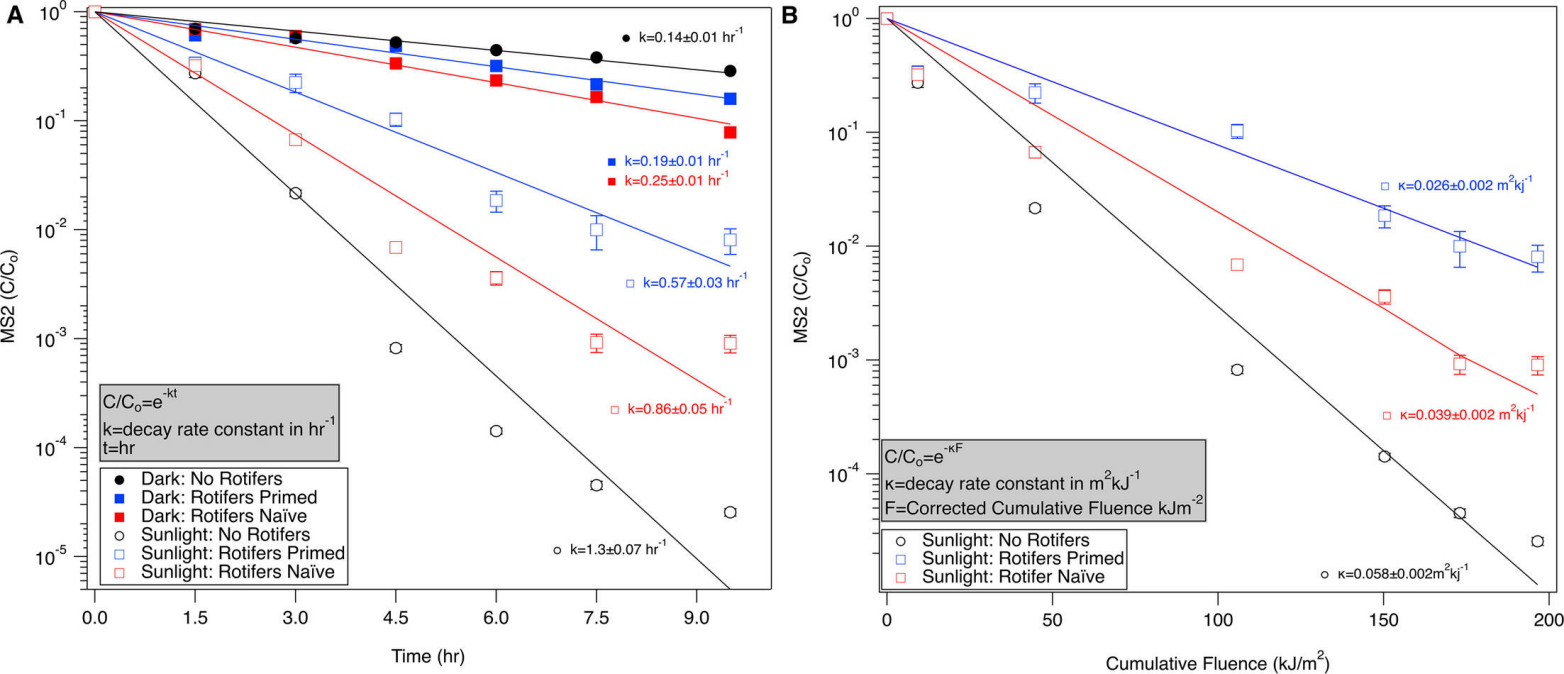
Effect of rotifers on inactivation of MS2 (A) as a function of time in the dark and light with and without rotifers and (B) as a function of cumulative fluence in the light with and without rotifers. Open symbols represent sunlight treatments, filled symbols represent dark treatments, and error bars represent the propagated error for triplicate treatments. Primed rotifers have been co-incubated with MS2 for 72 hours prior to sunlight exposure, while naïve rotifers have not. Lines represent fit for the first-order decay model for the inactivation rate constants, and the *r*^2^ values for sunlight treatments ranged from 0.93 to 0.95.

Based on the variability of inactivation rate constants in the literature, rate constants obtained from experimental treatments with rotifers were only compared directly to the inactivation rates obtained in the control treatments without rotifers in the same experiments. Live rotifer presence resulted in significantly less viral inactivation in sunlight compared to controls without rotifers (*P* < 0.05). Fig. 2 shows the inactivation curves as a function of time (Fig. 2A) and fluence (Fig. 2B). The lower inactivation rate constants (*k* or *κ*) for sunlight treatments containing rotifers are significantly different than the sunlight controls without rotifers, indicating protection effects from sunlight inactivation.

Two different rotifer treatments (primed and naïve) were deployed to test if primed rotifers, which had been acclimated to MS2 presence in the system and potentially already contained MS2 within their bodies, would have different effects on MS2 sunlight inactivation than naïve rotifers. While primed and naïve rotifers both provided significant protection of MS2 from sunlight in comparison to controls (*P* < 0.05), the two treatments did not significantly vary from each other (*P* = 0.065). The tissue data of viable virus recovered from rotifers, which is presented later in the manuscript, support the similar uptake and protection associated with primed versus naïve rotifers. However, the lack of statistical significance may not accurately represent biological significance or relevance. Comparison of the log removal values indicated a biologically significant difference between primed and naïve rotifers with approximately 1 log difference between the two experimental treatments by the end of 9.5 hours of sunlight exposure. More studies are needed to confirm if primed and naïve rotifers exhibit different protection effects. Exploring changes in feeding rate of rotifers may lead to a better understanding of potential differences between protection effects for naïve and primed rotifers.

Previous studies have shown that the feeding rate of rotifers is a function of food concentration, food type, and feeding history (52). Based on this difference in feeding history, the naïve rotifers and primed rotifers may have different ingestion rates as well as excretion rates. In addition, feces produced by rotifers may contain viable MS2, which may offer some protection from sunlight disinfection. While studies have shown that some particles can protect from disinfection efficiency, these studies did not directly test zooplankton feces (6, 18, 22). In our experiments, feces could not be separately collected or tested. Future experiments can be designed to help elucidate if there is a significant difference in protection effects for primed versus naïve rotifers and explore the factors described above. For example, long-term experiments can be conducted that compare clearance rates of MS2 or algal spikes at different concentrations and explore effects of feeding history. These experiments can be completed in conjunction with short-duration experiments to identify the gut passage time and ingestion rates specific to MS2.

### Dead rotifers contribute significantly less to protection against sunlight inactivation than live rotifers

**Fig 3:**
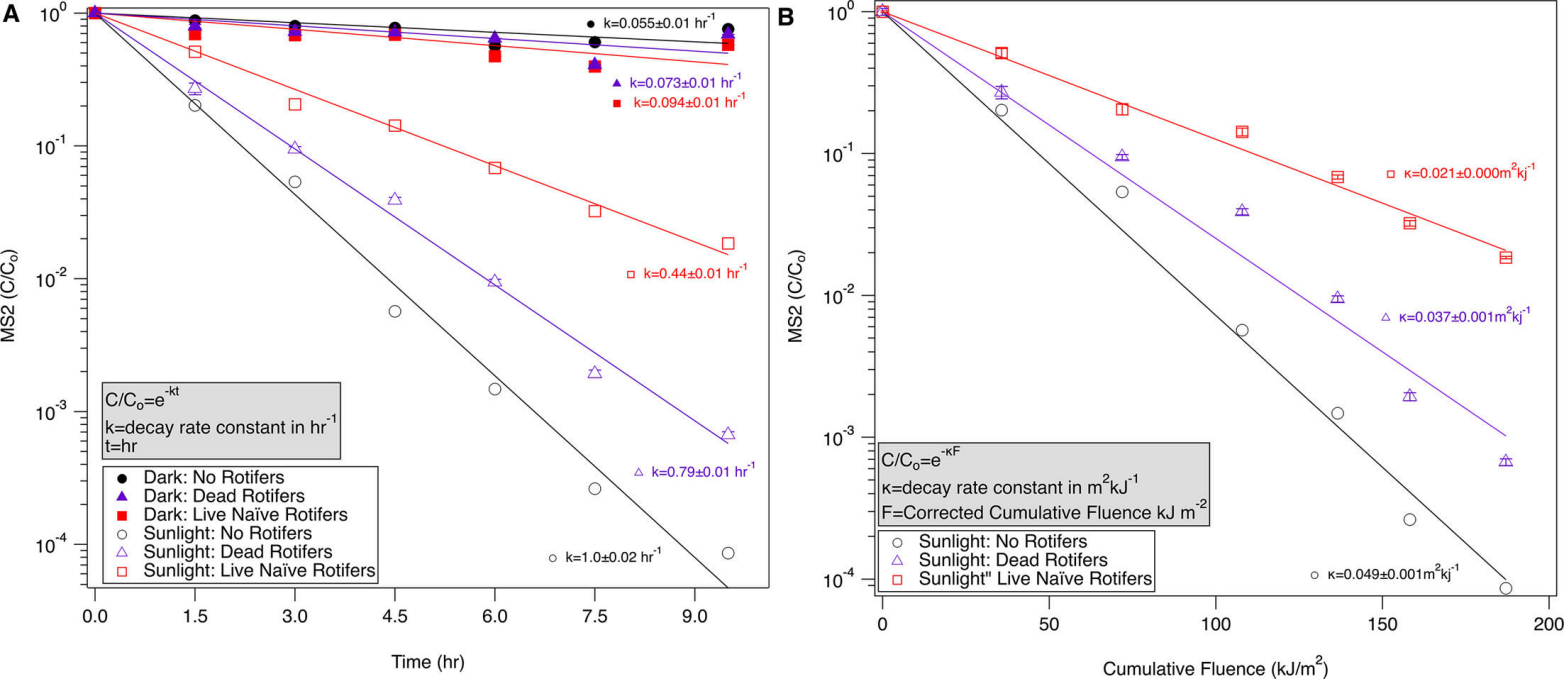
Comparison of live and dead rotifer effects on sunlight inactivation as a function of (A) time and (B) cumulative fluence. Dark controls are shown in Panel A for comparison. Open symbols represent sunlight treatments, filled symbols represent dark treatments, and error bars represent the propagated error for triplicate treatments. Primed rotifers have been co-incubated with MS2 for 72 hours prior to sunlight exposure, while naïve rotifers have not. Lines represent fit for the first-order decay model for the inactivation rate constants, and the *r*^2^ values for sunlight treatments range from 0.97 to 0.99.

To examine the contribution of the biological activity of live rotifers to protection effects from sunlight inactivation versus the contribution of shading and sorption effects of rotifers acting as particles, we also tested dead rotifers in sunlight exposure experiments (Fig. 3). While dead rotifers provide some protection in comparison to sunlight treatments without rotifers (*P* < 0.05), the protection effects were less pronounced in comparison to biologically active (live) rotifers. The inactivation rate constants (*k* and *κ*-values) obtained for sunlight treatments containing dead rotifers are significantly greater than those values from live rotifers (*P* < 0.05), indicating less protection effects. Due to the reduced overall solar irradiance on this experimental day versus the previous experiment with live rotifers (Fig. S3 and S4), utilizing the fluence-based *κ*-values provides a better comparison of live versus dead rotifers. The difference in calculated rates and observed decline in viral concentration between dead and live rotifers suggests that the biological activity of the live rotifers contributes significantly more to protection from sunlight inactivation than protection that may occur due to shading or sorption by dead rotifer bodies.

Dead rotifers in the system can likely be compared to protection observed by particles in other studies with sorption and shading being the dominant causes of such protection (53–55). With respect to testing sunlight protection of viruses by living organisms, studies have shown that enteric viruses internalized by the amoeba *Acanthamoeba sp*. are resistant to digestion, and amoebae can act as viral reservoirs that may resist disinfection (17, 23, 56). Only one study directly examines the impact of viral internalization by the free-living amoebae on UV disinfection and shows protection of internalized reovirus from UV (23). Bacteria ingested and internalized by nematodes, daphnids, and amoebae found in water treatment systems are also protected from various types of disinfection, including UV (57, 58). The cause of the protection effects of rotifers is not yet understood and requires further research. The only previously published study examining viral protection from UV by amoebae showed that the virus was internalized in the amoeba nucleus (23). Visualizing viral location may aid in understanding the cause of protection by rotifers, but it is outside the scope of this study.

Unlike amoebae, rotifers are filter-feeding organisms whose interaction with particulate matter changes based on system conditions. The filtration, clearance, and ingestion rate of rotifers can vary orders of magnitude for rotifers based on particle type (59–62). In our system, the algal clearance rate measured for rotifers varied from 0.01 to 0.1 ml hr^−1^ rotifer^−1^ during a 12 hour sampling period. The viral clearance rate is lower based on published literature showing that rotifers exhibit size-dependent clearance rates (59) and viruses are below the particle size range most efficiently filtered by rotifers. While the clearance rates provided in the literature and those calculated experimentally indicate that the rotifers in this experiment did not completely filter the entire content of the beakers during the experiment exposure time, they still had a significant impact on sunlight inactivation. The gut passage time*,* which ranges from 15 to 45 minutes for *Branchionus sp.* (62, 63), may have played a role in the protection effects seen with the rotifers. Based on our experimental observations, rotifer bodies may be somewhat impervious to sunlight, and the virus retained within the gut may be protected from inactivation and then excreted back into the system.

### Rotifers do not fully inactivate ingested virus

The virus was extracted from rotifer tissue after co-incubation experiments and sunlight exposure experiments. A viable virus was not recovered from dead rotifers that were co-incubated with MS2, confirming that our rinsing procedure removed sorbed virus from the rotifer body surface and that viral concentration results represent internalized amounts. Analysis of tissue from rotifers co-incubated from 12 to 120 hours without exposure to sunlight showed no significant relationship between viral concentration and incubation time. Concentrations from different co-incubation times were not significantly different (*P* > 0.05). These data indicate that rotifers retain a certain amount of virus in their gut. With a gut passage time of 15–45 minutes, the rotifers are likely excreting virus back into the system and then re-filtering water content that may have already passed through their gut.

**Fig 4:**
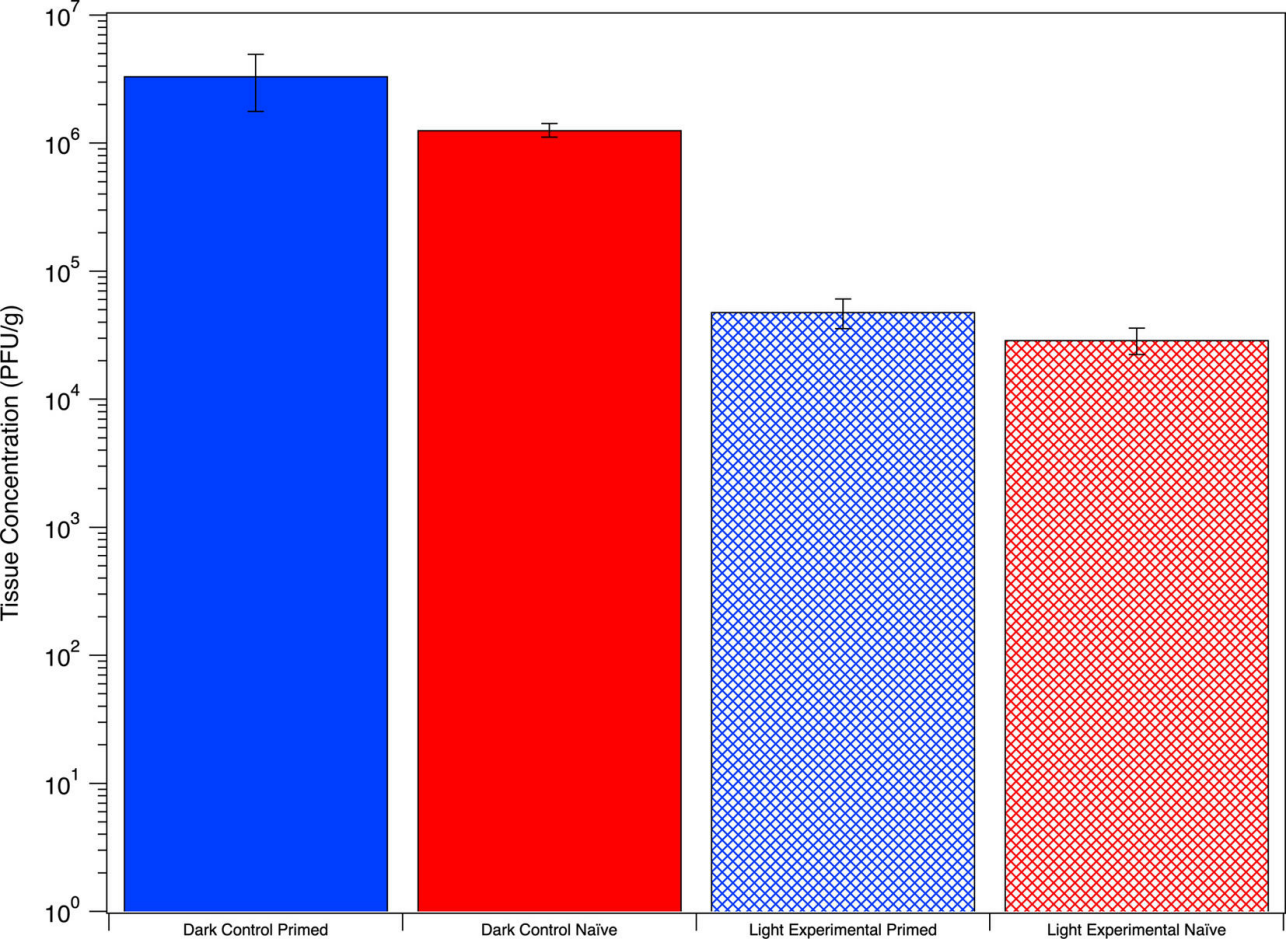
Concentration of viable MS2 recovered from rotifer bodies after sunlight exposure in comparison to dark experiments.

Similarly to co-incubation experiments, rotifers exposed to sunlight still contained viable viruses in their tissue, but the concentration was significantly lower (*P* < 0.05) than those in the dark, with a log difference of approximately 1.8.(Fig. 4) Naïve and primed rotifers had similar viral tissue loads, which aligns with the finding that exposure time has no impact on uptake rates and subsequently rotifer tissue viral loads. A material balance (in PFU), including media and tissue samples from the sunlight experiment, was completed to examine differences between initial and final amounts in the system (Fig. S6). The dark controls containing primed and naïve rotifers showed a 0.8 and 1.1 log decline, respectively, when comparing the initial and final time points. The light controls without rotifers showed an average 4.5 log decline from initial to final time point. Primed rotifers showed an average 2.1 log decline, and naïve rotifers showed a 3.0 log decline from initial and final time points. Close to 1 log difference was observed when comparing the primed and naïve rotifers, which aligns with the findings from the time series data with media (Fig. 2). Despite results from statistical tests, data indicate that primed rotifers exhibited biologically significantly more protection than naïve rotifers.

The statistically significant difference between dark and light rotifer tissue concentrations could indicate that sunlight does have some impact on viral inactivation. Examining the mechanisms and causes of this difference is an important research area requiring further experimentation. The absence of sunlight may have led to changes in rotifer feeding behavior and gut passage time. Studies have shown the importance of light intensity and wavelength on rotifer phototactic behavior (movement away or toward light) and population growth (64, 65), indicating that light availability impacts rotifer behavior. One possible explanation for the different viral concentration from dark versus light-exposed rotifer tissue is that the rotifers might have been filtering viruses inactivated by sunlight, leading to a lower concentration of retained viable viruses.

While experimentally determining the cause of the similar viral tissue loads in naïve and primed rotifers is outside of the scope of the current publication, the causes may once again be attributed to rotifer clearance rate and gut retention time of the virus. Studies have shown that the rotifer clearance rate can vary systematically with concentration of particles in the system (61, 66). Rotifers may only retain a certain concentration of virus, which may be related to studies showing that the rotifer clearance rate can be constant for a given particle up until an incipient limiting level, which then results in lower clearance rates (61, 66).

### Environmental significance

As filter-feeding organisms, rotifers play a critical role in natural and engineered aquatic systems by removing a variety of particles via filter feeding action and serving as prey for other organisms. Rotifers can reach high densities in aquatic systems and exert control on microbial populations (46, 47, 67). While the ability of rotifers to inactivate bacteria is documented (61), the results from this study show that rotifers can potentially act as a Trojan horse for viruses, limiting sunlight inactivation that can result in enhancing persistence. The ubiquitousness of rotifers combined with the protection effects from sunlight inactivation can lead to the dispersion of pathogens. Additional studies using different water matrices and virus types will provide a greater understanding of the water quality impact rotifers may have in an aquatic system. In addition, mechanistic studies are needed to understand why and how rotifers offer protection to viruses. After gaining a better understanding of rotifer impacts on viral disinfection, further studies involving multiple zooplankton species are needed to represent the complex trophic interactions in aquatic environments and help translate results to natural ecosystems.

## SUPPLEMENTAL MATERIAL

Supplemental material aem.02540-24-s0001.docx Figures S1 to S6.

ASM does not own the copyrights to Supplemental Material that may be linked to, or accessed through, an article. The authors have granted ASM a non-exclusive, world-wide license to publish the Supplemental Material files. Please contact the corresponding author directly for reuse.
