# Supplementary material for: Zooplankton protect viruses from sunlight disinfection
Source: Appl Environ Microbiol. 2025 Mar 31;91(4):e02540-24. doi: 10.1128/aem.02540-24 (PMC12016492; doi:10.1128/aem.02540-24)
Supplement: Supplemental material — Figures S1 to S6. [file aem.02540-24-s0001.docx]

**Supplemental Material**

**Zooplankton Protect Viruses from Sunlight Disinfection**

**Authors:** J.A. Wang^1^, O. Aryal^1^, L.N. Brownstein^1^, H. Shwwa^1^, A.L. Rickard^1^, A.E. Stephens^1+^, M. Lanzarini-Lopes^2^, N.S. Ismail^1*^

^1^Smith College Picker Engineering Program, ^2^University of Massachusetts Amherst Department of Civil and Environmental Engineering

*Address correspondence to Niveen S. Ismail, [nismail@smith.edu](mailto:nismail@smith.edu)

Number of pages: 6

Number of figures: 5


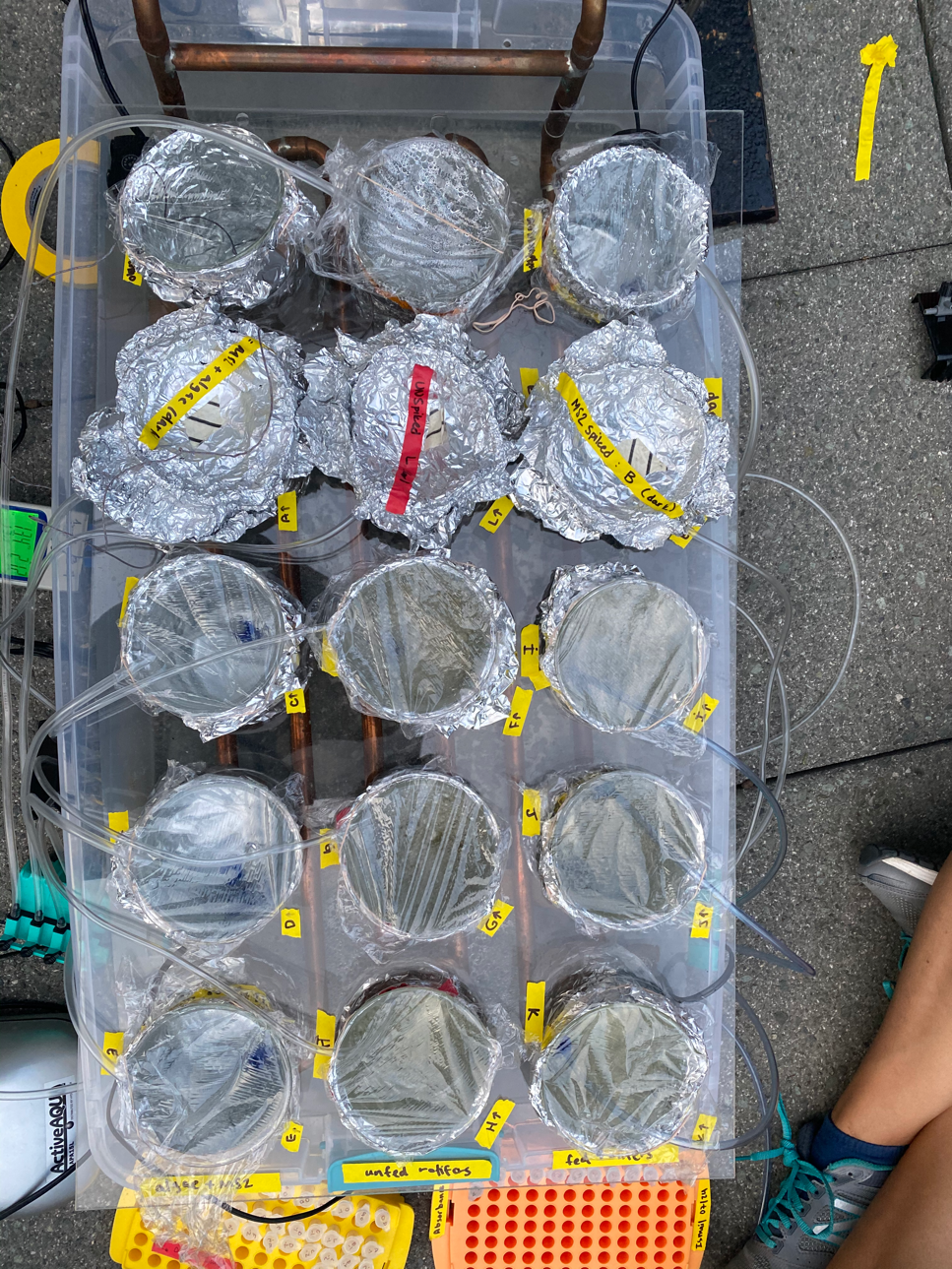

**Figure S1**: Example image and schematic of outdoor setup for sunlight inactivation testing


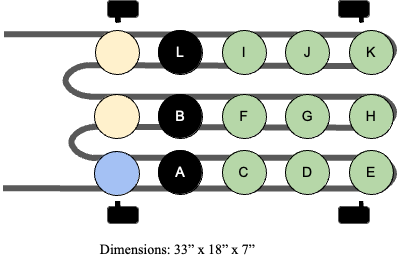


**Figure S2**: Example schematic of beaker microcosms in outdoor setup for sunlight inactivation testing

**Figure S3:** Solar spectra for two experimental days. Panel A solar spectra correspond to the experimental results for Figure 2. Panel B solar spectra correspond to the experimental results from Figure 3.

**Figure S4**: Comparison of normalized cumulative irradiance in arbitrary units for the two experimental days. Experiment 1 corresponds to the results from Figure 2. Experiment 2 corresponds to the results for Figure 3.

**Figure S5**: Representative absorbance values from UV-Vis with an integrating sphere.

**Figure S6:** Material balance from the sunlight inactivation experiment in PFU. Experimental treatments and light controls were completed in triplicate and plotted bars represent averages of each. Propagated error values are too small to visualize on the figure.
